# Amylose Inclusion Complexes as Emulsifiers for Garlic and Asafoetida Essential Oils for Mosquito Control

**DOI:** 10.3390/insects10100337

**Published:** 2019-10-11

**Authors:** Ephantus J. Muturi, William T. Hay, Robert W. Behle, Gordon W. Selling

**Affiliations:** 1 Crop Bioprotection Research Unit, Agricultural Research Service, U.S. Department of Agriculture, 1815 N. University St., Peoria, IL 61604, USA; Robert.Behle@ars.usda.gov; 2 Plant Polymer Research Unit, USDA, Agricultural Research Service, National Center for Agricultural Utilization Research, 1815 N. University St., Peoria, IL 61604, USA; William.Hay@ars.usda.gov (W.T.H.); Gordon.Selling@ars.usda.gov (G.W.S.)

**Keywords:** *Aedes aegypti*, essential oils, amylose inclusion complexes, emulsions

## Abstract

Although the insecticidal properties of some plant essential oils are well-documented, their use in integrated pest and vector management is complicated by their high volatility, low thermal stability, high sensitivity to oxidation, and low solubility in water. We investigated the use of bio-based *N*-1-hexadecylammonium chloride and sodium palmitate amylose inclusion complexes as emulsifiers for two essential oils, garlic and asafoetida, known to be highly toxic to mosquito larvae. Four emulsions of each essential oil based on amylose hexadecylammonium chloride and amylose sodium palmitate inclusion complexes were evaluated for their toxicity against *Aedes aegypti* L. larvae relative to bulk essential oils. All emulsions were significantly more toxic than the bulk essential oil with the lethal dosage ratios ranging from 1.09–1.30 relative to bulk essential oil. Droplet numbers ranged from 1.11 × 10^9^ to 9.55 × 10^9^ per mL and did not change significantly after a 6-month storage period. These findings demonstrated that amylose inclusion complexes enhanced the toxicity of essential oils and could be used to develop new essential oil based larvicides for use in integrated vector management.

## 1. Introduction

Essential oils are complex mixtures of volatile compounds produced by aromatic plants through secondary metabolic pathways. These oils serve as attractants for pollinators, contribute to plant defenses against herbivores and pathogens, and give plants their characteristic odors [1,2]. The aromatic and bioactive properties of essential oils have attracted great interest in their commercial production and applications in food processing, cosmetics, pharmaceutical, and textile industries [3]. Their low environmental toxicity, wide acceptance by consumers, and proven bioactivity against diverse insect pests and vectors has also stimulated research on their potential application as ecofriendly botanical insecticides [4,5,6,7,8]. For mosquito control, essential oils produce a wide range of biological effects ranging from direct lethal effects against all four life stages (i.e., eggs, larvae, pupae, and adult) to oviposition deterrent and repellent activity against adult mosquitoes [9,10,11,12,13,14]. These oils have emerged as a promising tool for combating insecticide resistance because they possess complex mixtures of bioactive compounds with different modes of action. They also can enhance the toxicity of synthetic insecticides [15,16,17].

Despite the well-documented insecticidal properties of some essential oils, their use in integrated pest and vector management is complicated by their high volatility, low thermal stability, and high sensitivity to oxidation [18,19]. In addition, the hydrophobic nature of essential oils makes them insoluble in water, causing non-uniform distribution in aquatic habitats. These characteristics reduce the effectiveness of essential oils in mosquito control when applied directly in aquatic habitats, and elevated concentrations that may be costly to apply and toxic to nontarget organisms are required to achieve meaningful larval control. Therefore, a delivery system that protects them from chemical degradation, improves their dispersion in water bodies, and extends their shelf life and activities is necessary to facilitate their practical use in insect pest and vector management [20]. 

Emulsion technology has received much attention as a promising delivery system for essential oils. Emulsions are heterogeneous systems consisting of two immiscible liquids (e.g., oil and water), where one of the liquids is dispersed as small droplets in another liquid and stabilized by a substance with emulsifying properties [21]. Emulsifiers adsorb at the interface between the two immiscible liquid phases imparting electrostatic or steric barriers between neighboring and colliding droplets, thus preventing coalescence. This unique characteristic of emulsifiers is attributed to their amphipathic nature, possessing both hydrophilic and hydrophobic groups in the same molecule, allowing them to interact with both the continuous and disperse phases of the system [22]. Emulsifiers are widely used to improve the stability and efficacy of essential oils in aqueous systems and to reduce the net concentration required to achieve the desired biological activity [23]. Emulsion breakdown, the separation of uniform dispersion into separate phases, can be inhibited by polymers, as polymers can provide steric stability from layers of the polymer chains to prevent oil droplet coalescence [24,25]. 

Starch is an abundant, renewable agricultural commodity that has received great interest as a raw material for application in emulsion technology [26]. This interest largely is due to its low cost and the presence of amylose, an essentially linear component of starch that, in the presence of complexing ligands such as fatty acids, long-chain alcohols, or monoglycerides, undergoes a conformational change to form a left-handed single helical amylose-ligand inclusion complex where the guest molecules are confined inside the helical cavity [27,28]. This amylose inclusion complex is often referred to as V-amylose because the amylose helix is hydrophilic on the outside and hydrophobic on the inside [29,30]. 

The ability of amylose to form helical structures and encapsulate compounds of interest is used in food, cosmetic, and pharmaceutical industries for delivery of various types of desirable bioactive compounds and contribute essential roles in improving food quality, extending shelf life of products, and enhancing drug uptake [29,31]. Amylose inclusion complexes have also been shown to be excellent emulsifiers for cedarwood essential oil for wood treatment against termites and wood-decay fungi [32]. Amylose-fatty acid salt inclusion complexes can be produced using conventional steam-jet cooking [33]. Unlike uncharged fatty acid inclusion complexes that form insoluble spherulites when cooled, amylose-fatty acid salt inclusion complexes are soluble in water, do not retrograde, and can be dried and easily re-dissolved in water [34,35]. The complexes formed from high amylose corn starch and fatty sodium salts (C12–C22) were found to be surface-active polymers with superior emulsion activity as compared with commercial octenyl succinic anhydride starch [36]. Amylose inclusion complexes capable of being used as emulsifiers have also been produced from high amylose corn starch and the phenolic aldehyde vanillin [37]. 

Amylose inclusion complexes are thought to effectively protect essential oils from oxidation, enhance their thermal stability, and improve their dispersion in aqueous phase [19]. These properties may reduce the concentrations of essential oils required to suppress mosquito larvae in aquatic habitats. The objective of this study was to investigate whether the use of amylose inclusion complexes as emulsifiers could improve essential oil delivery throughout the water column, prevent oil coalescence, and enhance toxicity against mosquito larvae. Garlic and asafoetida essential oils were chosen for this study because they are known to be highly toxic to mosquito larvae [38,39]. The findings of this study may inform the development and commercialization of essential oil based larvicides to complement other commercially available bio-larvicides such as *Bacillus thuringiensis* var *israelensis* (Bti). 

## 2. Materials and Methods

### 2.1. Preparation and Spray Drying of Amylose Complexes

High amylose corn starch (∼68% amylose, AmyloGel 03003) was a product of Cargill (Minneapolis, MN, USA). Sodium palmitate (98.5%), *N*-1-hexadecylamine (98%), and hydrochloric acid (HCl, 37%) were obtained from MilliporeSigma (St. Louis, MO, USA). Amylose complexes were produced following the procedure outlined previously [40]. A high-amylose starch dispersion prepared from 100.0 g (dry basis) of starch and 1800 mL of deionized water was passed through a Penick and Ford laboratory model continuous steam jet cooker (Penford Corp., Cedar Rapids, IA, USA). The jet cooker was operated under excess steam conditions with a steam line pressure of 70 psig. Cooking was carried out at 140 °C (40 psig steam within the hydroheater) with a pumping rate of about 1 L∙min^−1^. Cooked dispersions were collected in a Dewar flask to prevent rapid temperature loss. Solutions of sodium palmitate (prepared by dispersing 5.25 g of sodium palmitate in 217.42 g deionized water and heating to 90 °C) or *N*-1-hexadecylammonium chloride (prepared by dispersing 5.25 g of *N*-1-hexadecylamine in 217.42 g of 0.1 N HCl and heating to 90 °C) were added to the 90–95 °C dissolved starch solution. The solution was rapidly stirred for 1 min and then cooled in an ice bath to 25 °C. The cooled amylose complexes were isolated by spray drying using a Niro atomizer spray dryer (Niro, Columbia, MD, USA) as previously described [41]. Concurrent flow atomization of the feed stock was accomplished with a rotating disc atomizer at an operating pressure of 5.8 bar. Approximately 3.5% solids solution was fed at 30 mL∙min^−1^ with inlet temperatures of approximately 156 °C, which provided outlet temperatures of approximately 84 °C. Materials were collected and stored at room temperature until testing. 

### 2.2. Preparation of Emulsions

Oil-in-water (O/W) emulsions were produced using the amylose complex spray-dried powders as an emulsifier for garlic and asafoetida essential oils (New Directions Aromatics Inc., Mississauga, ON, Canada) in an aqueous continuous phase. The chemical compositions of the two essential oils were reported in our previous study, with allyl disulfide as one of the major compounds contributing to their toxicity [39]. Prior to emulsion formation, the amylose complexes were either dispersed as a dry powder in room-temperature water or dissolved in solution at 80 °C. Emulsions were formed at room temperature from a ratio of 92.5:5:2.5 of water, essential oil, and spray dried amylose complex emulsifier (where the complex was used either as a solution or as the dry powder), respectively. Essential oils, amylose complexes, and ultrapure water, totaling 10 g, were added to a 30 mL glass beaker and homogenized for 180 s at 20,000 rpm using a Power Gen 35 handheld micro homogenizer (Fisher Scientific, Pittsburgh, PA, USA).

### 2.3. Bioassays

Twenty late third instar larvae of *Aedes aegypti* were added into 120 mL of de-ionized (DI) water held in 400 mL tripour beakers. Treatments included garlic and asafoetida essential oils (New directions Aromatics, Mississauga, ON Canada) diluted in absolute ethanol (bulk essential oil) and four emulsions of each essential oil based on either amylose *N*-1-hexadecylammonium chloride (Hex-Am) or amylose sodium palmitate (Na-P) inclusion complexes either in solution or powder form. Two negative controls were used: an ethanol control and a corn oil Na-P emulsion. Each treatment was tested at 7 concentrations ranging from 2 to 16 ppm. The stock solution for bulk essential oil (50,000 ppm) was prepared by mixing 950 μL of absolute ethanol with 50 μL of either garlic or asafoetida essential oil. The control groups received either 38.4 μL of absolute ethanol without oil treatment or 38.4 μL of corn oil emulsion. This amount is equivalent to the volume of oil treatments used to achieve 16 ppm, the highest concentration used in this study. The experiment was replicated three times, and three separate trials were conducted. The containers were held at room temperature, and the total number of larvae surviving 24 h post-treatment was counted and recorded. Mortality data were analyzed using the R 3.3.2 statistical package [42]. Probit analysis was conducted using the ecotox package to determine the LC_50_ and LC_90_. To determine whether the LC_50_ values differed significantly between treatments, we calculated the lethal dosage ratios of emulsions and their 95% confidence limits relative to bulk essential oils. If these intervals did not include 1, the lethal concentrations of emulsions were considered to be significantly different from those of bulk essential oil [43]. 

### 2.4. Determination of Droplet Numbers and Diameter

Emulsion stability was evaluated for samples stored under refrigeration by determining the concentration of oil droplets based on hemocytometer counts, assuming that oil coalescence would result in fewer droplets. Garlic and asafoetida emulsions were inverted by hand 50 times to provide uniform suspensions. Each sample was then serial diluted 10×, 100×, and 1000× in DI water. The 1000× dilution of each emulsion was loaded onto a hemocytometer (Bright-line, Hausser Scientific, Horsham, PA, USA), and oil droplets were counted under a light microscope at 400×. Samples for 4 and all 8 emulsions, respectively, were counted when freshly prepared and after 6 months of storage. Droplet counts between fresh and stored samples were compared using a paired *t* test. To determine the particle size and distribution, dynamic light scattering (DLS) analysis was used. DLS analyses were carried out using a Horiba LB-550 Dynamic Light Scattering Particle-Size Analyzer (HORIBA Instruments Incorporated, Irvine, CA, USA) at 25 °C using a 1 cm path-length cell having a volume of 1.25 mL. Aqueous emulsions (minimum three samples tested) of the selected oils and amylose complexes were prepared as described and sufficiently diluted (~1000×) to obtain spectra. Hydrodynamic diameter distribution data were analyzed and processed to determine the median hydrodynamic diameter using the provided Horiba software. Intensity percent was obtained by determining the total area for each spectral curve and then for each diameter; its intensity percent was its value divided by the total area multiplied by 100.

## 3. Results

### 3.1. Toxicity of Essential Oil Emulsions

The toxicities of garlic and asafoetida essential oils were significantly higher when both Hex-Am and Na-P inclusion complexes were used as emulsifiers compared to bulk essential oil (Table 1). The LC_50_ values for garlic emulsions ranged from 5.97 ppm for Hex-Am solution to 7.24 ppm for Na-P powder compared to 7.95 ppm for bulk garlic essential oil (Table 1). The lethal dosage ratios for garlic emulsions ranged from 1.10 for Na-P powder to 1.33 for Hex-Am solution (Table 1). The corresponding LC_50_ values for asafoetida emulsions ranged from 8.11 ppm for Hex-Am solution to 9.90 ppm for Na-P powder compared to 10.57 ppm for bulk asafoetida essential oil. The lethal dosage ratios for asafoetida emulsions ranged from 1.07 of Na-P powder to 1.30 for Hex-Am solution (Table 1). Overall, for both essential oils, emulsions from solutions were more toxic than those based on dried powder.

### 3.2. Droplet Numbers, Size, and Distribution

**Figure 1:**
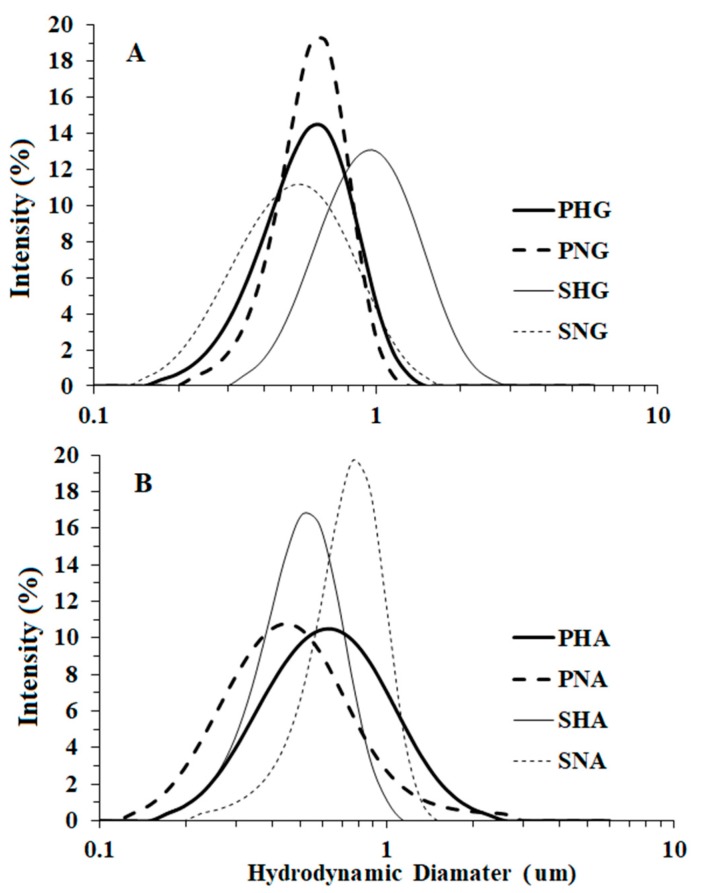
Representative dynamic light scattering analysis of the hydrodynamic diameter (μm) of (**A**) garlic and (**B**) asafoetida oil emulsions. PHG, garlic/amylose-Hex-Am powder; PNG, garlic/sodium palmitate powder; SHG, garlic/amylose-Hex-Am solution; SNG, garlic/sodium palmitate solution; PHA, asafoetida/amylose-Hex-Am powder; PNA, asafoetida/sodium palmitate powder; SHA, asafoetida/amylose-Hex-Am solution; SNA, asafoetida/sodium palmitate solution.

Droplet numbers ranged from 1.11 × 10^9^ to 9.55 × 10^9^ and did not change significantly over a 6-month storage period (Table 2). Emulsions from solutions had a greater number of oil droplets than those of dried powder. Also, Hex-Am emulsions had more droplet numbers than Na-P. Stored samples had no observable oil layer on the solution surface, but underwent droplet settling after 6 months. Simple agitation would resuspend the oil droplets forming a suspension. The median diameter of oil droplets for freshly prepared emulsions ranged from 0.42 ± 0.31 to 0.88 ± 0.37 μm (± SD), and their size distributions overlapped across treatments (Figure 1).

## 4. Discussion

The use of amylose-inclusion complexes as emulsifiers is appealing because they are bio-based, made from low-cost materials and processing, and are composed of materials that are generally regarded as safe, food-grade materials [36]. In this study, we investigated the use of Hex-Am and Na-P inclusion complexes as emulsifiers for essential oils to control mosquito larvae. These complexes were chosen since they are known emulsifiers formed using bound ligands with equivalent 16 carbon alkyl tails and possess differing cationic (Hex-AM) and anionic (Na-P) charges [32,36]. Aqueous emulsions of garlic and asafoetida essential oils were prepared using the two emulsifiers and their toxicity against mosquito larvae determined. Both essential oils are known to be highly toxic to mosquito larvae, as depicted by their low LC_50_ values [38,39], but their low solubility in water complicates their application in pest and vector management. The two emulsifiers enhanced the toxicity of both essential oils, demonstrating their potential application in the development of novel essential oil formulations for controlling mosquitoes and likely other arthropods of medical and economic significance. 

The high toxicity of aqueous emulsions of garlic and asafoetida essential oils relative to bulk essential oils may be due to their smaller droplet sizes. The emulsification of the essential oils allowed for a greater aqueous-phase concentration of the oils. Because essential oils are hydrophobic, they preferentially associate with and are encapsulated by the emulsifiers, allowing them to be dispersed in water at concentrations far greater than their typical solubility [23]. Stable emulsions were formed using spray-dried powders and dissolved aqueous solutions of the amylose complexes. The ability to form stable emulsions by simply applying shear to the formulation components lowers the processing costs and allows on-site production of the formulation when needed. Essential oil emulsions formed from solutions of the dissolved amylose complexes had the largest number of oil droplets and were more toxic than those formed using dried powders. Since all emulsions were produced using the same amount of materials, the number of droplets in a given sample volume should be relative to the efficacy of the emulsifier; the emulsifying activity is equivalent to the interfacial area of an emulsion, as the number of dispersed oil droplets increases as the interfacial area increases [44]. 

Mean droplet numbers ranged from 1.11 × 10^9^ for garlic/amylose-Hex-Am powder (PHG) to 9.55 × 10^9^ for garlic/amylose-Hex-Am solution (SHG), and median droplet diameters ranged from 0.42 μm for asafoetida/sodium palmitate powder (PNA) to 0.88 μm for SHG. Previous studies have shown that formulations with smaller droplet sizes have the highest toxicity [45]. Small droplet sizes provide a greater surface area, which improves their effective distribution in the water column and penetration into insect tissues [46]. In turn, these properties enhance the biological efficacy of essential oils and reduce the concentrations needed for pest and vector control applications. For example, the anticholinesterase inhibitory activity of microemulsions based on *Zingiber cassumunar* Roxburgh essential oil was 20 times higher than that of bulk *Zingiber cassumunar* essential oil [47]. The increased toxicity of smaller particles was not observed in this study, as both emulsifiers produced oil droplets of roughly the same size. Higher toxicity of Hex-AM emulsions relative to Na-P emulsions may be due to the higher number of oil particles or the composition of the complex. Additionally, the mosquito gut has an alkaline pH of approximately 11 [48], and the Hex-Am complex has been shown to dramatically gel at basic pH, as the ammonium chloride head group is neutralized to a primary amine [49]. This gelation may cause disruption in digestion, rapid release of the emulsified essential oil, and ultimately induce additional damage or stress to the insect, increasing the likelihood of mortality.

Emulsions are thermodynamically unstable, and keeping emulsion droplet sizes uniformly distributed during use and storage is a major concern because of destabilizing processes such as creaming, flocculation, and coalescence [50]. There are two general mechanisms of emulsion stabilization: electrostatic stability, where the repulsion among droplets due to high surface charge hinders droplet agglomeration, and steric stability, where the emulsifier molecule adheres to the oil droplet surface [51,52]. Electrosteric stability can also occur where both types of stabilization are observed. The 4 emulsions whose oil droplets were counted both when freshly prepared and after a 6-month storage period revealed no significant difference in droplet numbers between the two time points. These findings suggest that at least one of the stabilizing processes was in place preventing the emulsions from creaming, flocculation, or coalescence. Emulsions formed from amylose fatty sodium salt complexes were previously observed to have highly stable droplet sizes with no significantly observed coalescence [36]. The mechanism of emulsion formation is unclear, but the amylose complexes may form a pickering-like emulsion from microphase-separated structures similar to Janus particles or block copolymers [37,53,54]. This is reinforced by the extradordinary stability of the essential oil emulsions, a typical feature of pickering emulsions, and the lack of coalesence observed in the oil droplets. 

Currently, there is increased interest in the discovery and development of ecofriendly biopesticides that can also combat the rapidly emerging problem of insecticide resistance. Essential oils of some plants meet these criteria because they have low mammalian toxicity, degrade quickly in soil and water, possess ovicidal, larvicidal, adulticidal, and repellent activity against mosquitoes, and their multiple modes of action and sites of action make it difficult for mosquitoes to develop resistance [7,9,10,11,12,13,14,55,56,57]. Recently, we observed that both garlic and asafoetida essential oils have strong ovicidal and larvicidal activities against mosquito larvae, justifying their choice for the current study [39]. Several studies have investigated the potential application of essential oil based emulsions in mosquito control. An attractive sugar bait consisting of 0.4% beta-cyclodextrin microencapsulated garlic essential oil and 99.6% mixture of date syrup, citrus juice, sucrose, and water was highly effective against the Asian tiger mosquito, *A. albopictus* Skuse [58]. Exposure of *A. aegypti* L. larvae to nanoemulsion consisting of 90% water (*w*/*w*), 5% *Rosmarinus officinalis* L. essential oil (*w*/*w*), and 5% (*w*/*w*) polysorbate 20 (nonionic surfactant) at 250 ppm concentration resulted in 80% and 90% mortality after 24 and 48 h, respectively [59]. A 100% mortality of *A. aegypti* larvae was observed following exposure to 250 ppm of nanoemulsion consisting of 5% (*w*/*w*) *Pterodon emarginatus* Vogel essential oil, 5% (*w*/*w*) surfactant (polysorbate 80), and 90% (*w*/*w*) water [60]. At 250 ppm concentration, nanoemulsion consisting of 6% (*v*/*v*) eucalyptus essential oil, 12% tween 80 (nonionic surfactant), and 82% water had rapid larvicidal activity against *Culex quinquefasciatus* Say, killing 98% of the larvae within 4 h of treatment compared to bulk essential oil, which caused 100% mortality after 24 h [61]. Microemulsions of geranium essential oil were more toxic to *C. pipiens* Linnaeus larvae compared to bulk geranium essential oil (LC_50_ = 32 vs. 95.94 ppm) [45]. These findings demonstrate that essential oil formulations could indeed serve as effective biopesticides for mosquito control. Our garlic and asafoetida oil emulsions are especially promising because they are more lethal than the formulations described above. 

Among the few biopesticides that have been commercialized, Bti is the most widely used because of its high potency and specificity [62]. For example, the LC_50_ values for Bti against *A. aegypti* larvae range from 0.04–0.7 ppm, suggesting it is more toxic than our garlic and asafoetida emulsions [63,64]. However, evolution of Bti resistance remains a serious concern highlighting the need for the discovery and development of new biopesticides for application in integrated vector management [65,66,67]. Additionally, some essential oils have been shown to work in synergy with Bti [68]; thus, a combination of Bti and essential oil emulsions may be a novel strategy for managing insecticide resistance. Further studies are needed to determine how garlic and asafoetida essential oil emulsions interact with Bti and other commercially available biopesticides. 

## 5. Conclusions

This study shows that emulsions based on garlic and asafoetida essential oils are highly toxic to mosquito larvae and could be an effective and ecofriendly strategy for controlling vector mosquitoes. The amylose complexes are bio-based emulsifiers formed from low-cost materials in an industrially relevant method. The Hex-AM emulsions provided more oil particles and was more lethal than the Na-P emulsions; some amount of the increased lethality would be attributable to the smaller particle size. An improved relative potency of the treatments would lead to a cost reduction of the final treatment application, as the essential oil represents most of the material expense. There is need for additional studies to determine the nontarget effects of these emulsions and to assess and optimize their stability under diverse ecological settings before their commercial application in mosquito control. Additionally, cationic surfactants such as Hex-Am inclusion complexes have antimicrobial properties [32,69], and it is desirable to investigate their influence on microbial communities in aquatic habitats and on mosquito-associated microbiota. The latter are being investigated for potential application in symbiotic control of mosquito-borne diseases, and their study could reveal important synergies that could be tapped to improve the success of vector control programs.

## Figures and Tables

**Table 1:** LC_50_ values for garlic and asafoetida essential oil emulsions relative to bulk essential oil. Amylose-Hex-Am, Amylose-hexadecylammonium chloride inclusion complex. LDRs = lethal dose ratios.

Treatment	Label	n	LC50 (95% CI)	Chi-Square	LDRs at LC50 (95% CI)
Garlic	Garlic	180	7.95 (7.19–8.66)	29.84	
Garlic/Amylose-Hex-Am powder	PHG	180	6.62 (6.23–6.98)	5.63	1.20 (1.14–1.26)
Garlic/Sodium palmitate powder	PNG	180	7.24 (6.31–8.08)	22.40	1.10 (1.04–1.16)
Garlic/Amylose-Hex-Am solution	SHG	180	5.97 (5.68–6.24)	4.18	1.33 (1.26–1.41)
Garlic/Sodium palmitate solution	SNG	180	6.29 (5.70–6.83)	7.79	1.26 (1.19–1.34)
Asafoetida	Asafoetida	180	10.57 (8.74–12.88)	81.68	
Asafoetida/Amylose-Hex-Am powder	PHA	180	9.03 (8.53–9.51)	8.66	1.17 (1.12–1.22)
Asafoetida/Sodium palmitate powder	PNA	180	9.90 (9.15–10.62)	14.78	1.07 (1.02–1.12)
Asafoetida/Amylose-Hex-Am solution	SHA	180	8.11 (7.67–8.54)	8.75	1.30 (1.25–1.36)
Asafoetida/Sodium palmitate solution	SNA	180	9.43 (8.99–9.88)	5.12	1.12 (1.07–1.18)

**Table 2:** Droplet numbers of garlic and asafoetida essential oil emulsions. Samples were diluted in de-ionized water at 1:1000. Dash (-) indicates that droplet numbers were not determined.

Treatment	Label	Concentration (Particles/mL) (02/21/18)	Concentration (Particles/mL) (8/17/18)
Garlic/Amylose-Hex-Am powder	PHG	1.11 × 10^9^	3.22 × 10^9^
Garlic/Sodium palmitate powder	PNG	2.52 × 10^9^	2.54 × 10^9^
Garlic/Amylose-Hex-Am solution	SHG	-	9.55 × 10^9^
Garlic/Sodium palmitate solution	SNG	-	3.48 × 10^9^
Asafoetida/Amylose-Hex-Am powder	PHA	1.79 × 10^9^	4.18 × 10^9^
Asafoetida/Sodium palmitate powder	PNA	3.96 × 10^9^	2.33 × 10^9^
Asafoetida/Amylose-Hex-Am solution	SHA	-	4.34 × 10^9^
Asafoetida/Sodium palmitate solution	SNA	-	2.73 × 10^9^
